# Effect of two different tooth bleaching techniques on microhardness of giomer

**DOI:** 10.4317/jced.53290

**Published:** 2017-02-01

**Authors:** Soodabeh Kimyai, Mahmoud Bahari, Fereshteh Naser-Alavi, Soodabeh Behboodi

**Affiliations:** 1 Dental and Periodontal Research Center, Faculty of Dentistry, Tabriz University of Medical Sciences, Tabriz, Iran; 2 Professor, Department of Operative Dentistry, Faculty of Dentistry, Tabriz University of Medical Sciences, Tabriz, Iran; 3 Assistant Professor, Department of Operative Dentistry, Faculty of Dentistry, Tabriz University of Medical Sciences, Tabriz, Iran; 4 Post graduate student, Department of Operative Dentistry, Faculty of Dentistry, Tabriz University of Medical Sciences, Tabriz, Iran; 5 Under graduate student, Department of Operative Dentistry, Faculty of Dentistry, Tabriz University of Medical Sciences, Tabriz, Iran

## Abstract

**Background:**

Tooth bleaching is a safe and conservative treatment modality to improve the esthetic appearance of discolored teeth. One of the problems with the use of bleaching agents is their possible effect on surface microhardness of resin-based materials. The present study was carried out to evaluate the effect of in-office and at-home bleaching on surface microhardness of giomer.

**Material and Methods:**

Seventy-five disk-shaped giomer samples (Beautifil II) were prepared and cured with a light-curing unit. The samples were randomly assigned to three groups (n=25). In group 1 (control), the samples were stored in distilled water for 14 days. The samples in groups 2 and 3 underwent a bleaching procedure with 15% carbamide peroxide (CP) (8 hours daily) and 45% CP (30 minutes daily), respectively, for 14 days. Finally, the microhardness of samples was measured with Vickers hardness tester using a 100-g force for 20 seconds. One-way ANOVA was used to compare the mean microhardness values among the study groups, followed by post hoc Tukey test for two-by-two comparison of the groups. Statistical significance was set at *P*<0.05.

**Results:**

One-way ANOVA showed significant differences in the mean microhardness values among the study groups (*P*<0.001). Based on the results of Tukey test, microhardness in the bleached groups was significantly less than that in the control group (*P*<0.0005). In addition, microhardness in the 45% CP group was significantly less than that in the 15% CP group (*P*<0.0005).

**Conclusions:**

Use of both bleaching agents during in-office and at-home bleaching techniques resulted in a decrease in surface microhardness of giomer. The unfavorable effect of in-office bleaching (45% CP) was greater than that of at-home bleaching (15% CP).

** Key words:**Dental restorations, hardness, tooth bleaching.

## Introduction

Achieving favorable and esthetic restorations is one of major concerns for dentists (1). Although the esthetic appearance of discolored teeth can be improved with many techniques, bleaching is a safe, conservative, low-cost and effective technique (2). Different bleaching agents have been marketed; however, the most commonly used active material is carbamide peroxide (CP) (3).

During tooth bleaching, free radicals of peroxide are deposited in the crystalline structure of enamel and oxidize the precipitated dyes, resulting in tooth whitening (4). Tooth bleaching is carried out either at home or in the office (5). During the in-office technique, the bleaching agents used consist of 30-35% hydrogen peroxide (HP) or CP applied during 15-60-minute periods on tooth surfaces based on the manufacturer’s instructions (6). For at-home bleaching technique, a special tray is fabricated for the patient and the patient places the bleaching gel containing 10-16% CP for 4-8 hours daily in the tray in contact with the teeth for 2-4 weeks (7).

Patients seeking tooth bleaching have amalgam and composite resin restorations in their teeth in many cases. Composite resins are more susceptible to chemical changes, compared to the neutral metallic and ceramic restorative materials, due to the presence of an organic matrix in their chemical structure (8). Different studies have yielded contradictory results in relation to the effect of bleaching gel on surface microhardness of composite resins (9-11). Taher reported a decrease in surface microhardness (9) and Mujdeci *et al.* reported an increase in composite resin surface microhardness subsequent to the use of bleaching agents (10). However, Polydorou *et al.* did not report any changes in this parameter with the use of bleaching gel (11).

Giomers are a new group of direct, adhesive restorative materials that exhibit esthetic, handling and physical characteristics of composite resins in association with advantages such as high radiopacity, an anti-plaque effect, and release and recharge of fluoride. These hybrid esthetic restorative materials are manufactured based on the pre-reacted glass-ionomer (PRG) technology and constitute the stable phase of glass-ionomer in the restorative material (12). Giomers exhibit better surface finish than conventional glass-ionomers and resin-modified cements, and their finish is comparable to that of composite resins and compomers (13).

Since the effect of bleaching agents has not been evaluated on the microhardness of giomers to date, the present study was undertaken to evaluate the effect of at-home and in-office bleaching techniques on the microhardness of giomers.

## Material and Methods

Beautifil II (Shofu Inc., Kyoto, Japan) with A2 shade was used in the present *in vitro* study. A total of 75 disk-shaped giomer samples were prepared using a round mold measuring 2 mm in thickness and 10 mm in diameter. The round mold was placed on a glass slab and after placing giomer within the mold, a transparent matrix strip (Hawe Neos Dental, Bioggio, Switzerland) was pressed on the mold to create a smooth surface and prevent an oxygen-inhibited layer.

The giomer samples were cured with the use of a QTH light-curing unit (Astralis 7, Ivoclar Vivadent, FL 9494, Schaan, Liechtenstein) at a light intensity of 400 mW/cm2 through the transparent matrix strip without any space in between, for 20 seconds. The supper surface of the samples was determined and the samples were polished with medium, fine and superfine polishing disks (Soflex, 3M ESPE, St. Paul, USA), followed by rinsing with distilled water. Then the samples were placed in an ultrasonic cleaner for 3 minutes to remove all surface debris (14). Finally, the samples were stored in distilled water at 37°C for 24 hours. The samples were subsequently assigned to 3 groups randomly (n=25).

Group 1 (control): The samples were stored in distilled water at 37°C for 14 days.

Group 2: The samples were subjected to a bleaching procedure with 15% CP (Opalescence® PF Ultradent Products, South Jordan, UT, USA) for 8 hours a day for 14 days.

Group 3: The samples were subjected to a bleaching procedure with 45% CP (Opalescence® Quick PF, Ultradent Products, South Jordan, UT, USA) for 30 minutes a day for 14 days.

**Figure 1:**
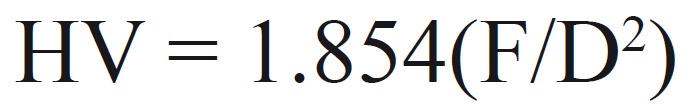
Formula.

The bleaching agent was placed on the tooth surface so that the entire surface was covered with an adequate amount of the bleaching agent. After the bleaching procedure, the samples were rinsed with distilled water and stored in distilled water at room temperature until the next procedure. In all the groups, fresh distilled water was used each day (14). It should be pointed out that the bleaching procedures in all the groups were carried out based on the instructions provided by the manufacturers. Vickers hardness testing machine (Walter Uhl, Aßlar, Germany) was used to determine the surface microhardness of the samples in three groups. The Vickers indenter was placed on the surface of each sample at room temperature for 20 seconds using a 100-g force (14). To this end, the giomer samples were dried with a piece of gauze and their upper surface was placed under the indenter of the machine. These indentations were made at a 1-mm distance from the margins and other indentations were created randomly (14). Microhardness values were calculated at 3 points by measuring the diameter of the rhomboid indentation using the following formula (10): (Fig. 1).

Then the mean of the 3 points was calculated as surface microhardness of each sample. In the present study, two samples were selected from each group for ultrastructural and surface topography evaluations under a scanning electron microscope (SEM; CamScan MV2300, Brno, Czech Republic) at ×5000 after covering the surface with a thin layer of gold.

One-way ANOVA was used to compare the means of microhardness values among the study groups. Then post hoc Tukey test was used for two-by-two comparison of the groups. Normal distribution of data was analyzed with Kolmogorov-Smirnov test and Levene’s test was used to evaluate homogeneity of variances among the groups. Statistical significance was set at *P*<0.05.

## Results

**Figure 2:**
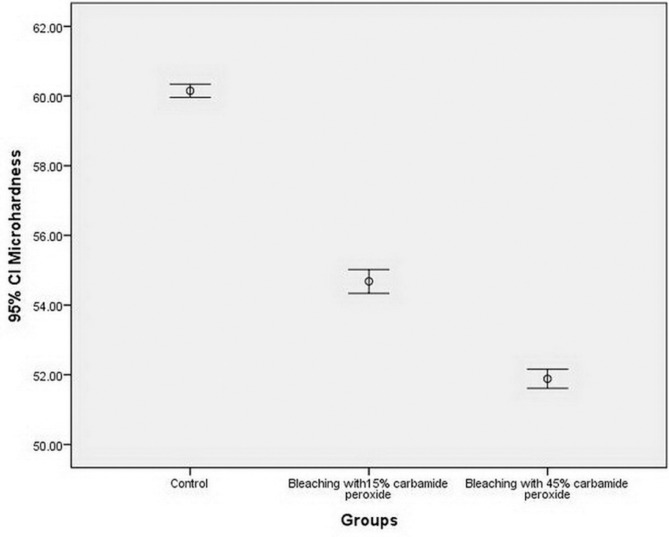
Error bar graph of the mean microhardness values in the study groups.

In groups 1-3 the means and standard deviations of microhardness values were 60.15±0.46, 54.67±0.82 and 51.88±0.65, respectively. Figure 2 shows the error bar graph of the mean microhardness values in the study groups. The results of one-way ANOVA showed significant differences in the mean surface microhardness values among the study groups (F72و2=990.427, *P*<0.001). Based on the results of Tukey tests, the microhardness in the control group was significantly higher than that in the 15% and 45% CP groups (*P*<0.0005). In addition, surface microhardness in the 15% CP group was significantly higher than that in the 45% CP group (*P*<0.0005).

**Figure 3:**
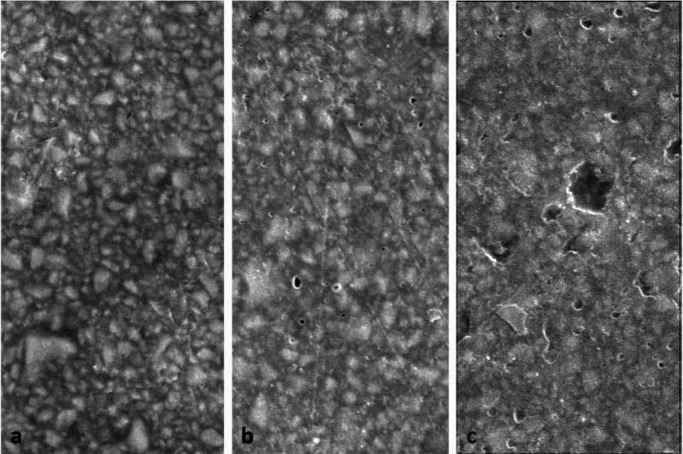
Evaluation of giomer samples under a scanning electron microscope at ×5000: a) the control group; b) 15% CP bleaching group; c) 45% CP bleaching group.

Figure 3 represents SEM micrographs of the selected samples in the study groups. Subjective evaluation of surface topography of the groups showed some changes and loss of some resin in the samples undergoing bleaching. It appeared the changes in the 45% CP group were greater than those in the 15% CP group.

## Discussion

Since surface hardness reflects the compressive strength and wear resistance and is one of the most important physical properties of resin-based materials, the present study evaluated the effect of tooth bleaching with 15% and 45% CP on surface microhardness of giomer. Based on the results, surface microhardness in the giomer groups undergoing bleaching was significantly less than that in the control group.

In this context, Kamangar *et al.* evaluated the effects of 15% CP and 40% HP bleaching agents and reported a decrease in microhardness of composite resin after application of these materials (14). In a study by Taher, too, application of 15% CP in an at-home bleaching technique resulted in surface softening of composite resin (9). Generally, CP is disintegrated into 1/3 hydrogen peroxide and 2/3 urea (15). The hydrogen peroxide produced after the application of a bleaching agent is very unstable and breaks the double bonds and separates the polymer chains by producing free radicals, which might have a relationship with a decrease in microhardness. The free radicals affect the resin-filler interface, creating microcracks (16). Therefore, bleaching agents can affect both the resin matrix and the filler-matrix interface; however, they do not affect the filler particles (17).

In the present study, 45% CP resulted in a greater decrease in giomer microhardness compared to 15% CP, which might be attributed to the fact that higher concentrations of carbamide peroxide gel release more hydrogen peroxide, resulting in an increase in disintegration of resin and a greater decrease in microhardness (18,19).

Contrary to the results of the present study, Polydorou *et al.* did not report any decrease in the microhardness of composite resin after evaluation of the effect of at-home bleaching technique (15% CP) (11) and in-office bleaching (38% HP) (20). Yu *et al.*, too, showed no changes in the microhardness of composite resin after the application of 15% CP (21).

The differences in the results of different studies might be attributed to differences in methodology, type and concentration of bleaching agents and the type of the substrate. In previous studies, different types of composite resin have been evaluated; however, in the present study, the substrate was giomer (Beautifil II). In contrast to composite resins that contain inorganic fillers, the filler particles in giomer originate from the pre-reacted glass-ionomer (PRG) technology. As mentioned above, bleaching agents do not affect inorganic filler particles, which might result in different responses in different substrates.

Subjective assessment of the samples showed porosity and loss of a small amount of resin in samples undergoing bleaching. It appears changes in the 45% CP group were more than those in the 15% CP group. These changes might indicate a real separation of the filler phase from the matrix. Pruthi *et al.*, too, reported an increase in composite resin roughness after treatment with 15% CP with the use of SEM evaluations (22). Smooth surfaces increase esthetic appearance and prevent formation of biofilms and accumulation of plaque; they might also decrease wear rate (23). During the bleaching process, the free peroxide radicals, apart from softening the resin matrix and decreasing microhardness, might affect the resin-filler interface, resulting in debonding of filler-matrix. This might be associated with microscopic cracks and an increase in surface roughness on SEM images (24). However, Wattanapayungkul *et al.* reported no changes in the roughness of composite resins with the application of bleaching materials; in this context, bleaching resulted in an increase in surface roughness in polyacid-modified composite resin materials (25). Lopes *et al.*, too, did not report any changes in surface morphology with the use of 10% CP at-home bleaching (26).

The effect of bleaching agents on the surface characteristics of materials depends on the bleaching agent, the duration of application and the type of the substrate (20). In this context, composite resins with a higher volume of organic matrix, such as microfilled composite resins, are more susceptible to the detrimental effects of bleaching agents, and SEM evaluations reveal cracks between the resin matrix and pre-polymerized particles (27). Although previous studies have attributed the negative effects of bleaching agents to the pH of the gel and have reported that usually the structural changes in the substrate occur at pH values <5.2 (28), in the present study, changes in microhardness and morphology of the substrate occurred at an approximate pH value of 6.5 of the bleaching gel.

It is difficult to extend the results above to clinical conditions because in the oral cavity, higher temperatures and constant contact with saliva might accelerate disintegration, decreasing the effects of the materials. It is suggested that studies be carried out under conditions closer to *in vivo* conditions and bleaching agents be evaluated at different concentrations and with the use of other bleaching agents such as hydrogen peroxide with different pH values.

Under the limitations of the present study, application of both in-office and at-home bleaching agents resulted in a decrease in surface microhardness of giomer. The detrimental effect of in-office technique with 45% CP was higher than that of at-home technique with 15% CP.
